# Improving public health through the development of local scientific capacity and training in rehabilitation in LMICs: A proof-of-concept of collaborative efforts in Parakou, Benin

**DOI:** 10.3389/fpubh.2022.952727

**Published:** 2022-09-07

**Authors:** Bruno Bonnechère, Oyéné Kossi, Thierry Adoukonou, Karin Coninx, Annemie Spooren, Peter Feys

**Affiliations:** ^1^REVAL Rehabilitation Research Center, Faculty of Rehabilitation Sciences, Hasselt University, Diepenbeek, Belgium; ^2^ENATSE, National School of Public Health and Epidemiology, University of Parakou, Parakou, Benin; ^3^Human-Computer Interaction and eHealth, Hasselt University, Diepenbeek, Belgium

**Keywords:** rehabilitation, LMICs, public health, EBP, WHO

## Rehabilitation services: A key player in the public health

The increase in life expectancy is linked, among other factors such as genetic factors, environmental factors, factors of medical conditions, socio-demographic factors, self-management, and access to care, to an increase in non-communicable diseases (NCDs) (1), which puts huge pressure on the health care systems. Cardiovascular diseases are, by far, the most common NCD in low- to middle-income countries (LMICs) (2); among these diseases, stroke is the most frequent (3). To face the huge public health challenge of NCDs and to lower their associated burden for patients and their families and caregivers, rehabilitation services are an essential part of management of these diseases (4). Rehabilitation is a set of interventions designed to optimize functioning and reduce disability in individuals with health conditions in interaction with their environment. Rehabilitation can be provided in many different settings, from the conventional inpatient or outpatient hospital setting to private clinics or in community settings, such as an individual's home. The rehabilitation workforce is made up of different health workers, including but not limited to physiotherapists, occupational therapists, speech/language therapists, audiologists, orthotists, and prosthetists, clinical psychologists, physical medicine and rehabilitation doctors, and rehabilitation nurses (5). Evidence-based practice (EBP) in rehabilitation has been defined by Novak et al. as “the cornerstone of care to maximize client outcomes through the application of best available interventions” (6). It must be noted that interventions are not limited to care but also included promotion, prevention, and diagnosis (7). According to the WHO, managing disability should be a priority in healthcare [i.e., Rehabilitation Action Plan 2030 (4)]. Verily, the demand for rehabilitation care is increasing, but there are human and financial constraints in the delivery of rehabilitation services. This situation is particularly true for patients living in LMICs (8). Health is a human right and if human rights are “rights held by individuals simply because they are part of the human species,” then all people, regardless of geography, should be entitled to the same collective efforts that can protect or improve their health (9). Unfortunately, there are huge disparities in access to rehabilitation services in LMICs.

Benin is currently facing the double burden of acute and chronic diseases with only limited resources allocated to the healthcare system (10). However, the clinical management and healthcare policy are still mainly oriented to treat infectious disorders. NCDs result in significant disability and decreased function and the quality of life of patients. The UN Human Development Program categorizes Benin as a “low human development” country, with a ranking of 158 out of 189 countries on the list. It is paramount to allocate sufficient resources for rehabilitation care in Benin to reduce the burden of disability after NCDs and to maximize the benefit of the neurorehabilitation program (11). It is therefore crucial to use EBP to strive for efficient use of resources.

According to the WHO, one of the most significant constraints on the rehabilitation process is lack of access to specialist facilities or healthcare personnel (5). The use of mobile technologies and eHealth may provide an alternative to the above-mentioned restrictions of rehabilitation or may supplement the present rehabilitation programs. The development and deployment of mHealth expands the healthcare sector's views and potential, at least in high-income countries (12–16). mHealth has opportunities for being a low-cost option with the potential to overcome the need for rapid access to healthcare clinics. Especially in LMICs, we think that this technology might contribute to reduce the lack of healthcare professionals, advance rehabilitation, and minimize health inequalities by improving the access to rehabilitation either remotely (due to scarcity of facilities particularly in rural regions) or financially.

In the remainder of this article, we highlight our strategy and collaborative efforts regarding the implementation of affordable technology-supported rehabilitation services, including mHealth, in the region of Parakou, Benin, with the aim to improve individual and public health.

## The main challenges of EBP in LMICs

Despite the obvious need for EBP in LMICs, the development and implementation of EBP are currently applied more in HICs (17). This disparity is due to multiple barriers that limit its application in LMICs.

The first challenge is the lack of training programs. In fact, the only university-level education of physiotherapy (PT) in all of French-speaking western Africa is in Benin (bachelor and master levels). This physiotherapy program was established in 2000 in Cotonou (*Ecole Supérieure de Kinésithérapie*) thanks to the support of the so-called B4 initiative (collaboration of Belgium with Benin, Burundi, and Burkina Faso). However, since there is a lack of training in other French-speaking African countries, more than half of students who graduated (approximately 20 students are graduating every year) are international students who return to practice in their country after their training; therefore, the number of graduates, as reported by the Benin Physiotherapy Association, is still very low approximately 220 PT are working in Benin, among them 97 are member of the association (18). The workforce of physiotherapy specializing in neurorehabilitation is only a very low fraction of that (*n* = 6). There is thus an urgent need to develop more training facilities in western African countries to catch up to the level of training offered in Benin. It is also of utmost importance to create a training program for other rehabilitation discipline, as the number of other specialists is poor, i.e., there is no educational program for occupational therapy (19) or occupational therapists in the country or for speech therapists (20). There is also a lack of neuropsychologists (21) and medical doctors specializing in physical medicine and rehabilitation, although they play an important role in the management of stroke. Another limitation is the paucity of knowledge (i.e., health literacy) of the population about the content of rehabilitation (22).

The second limitation that may explain a relatively low implementation of EBP–in LMICs–is the difficulty of translation of the research and the evidence from the well-developed rehabilitation services (e.g., infrastructure, reimbursement, integration in the care) in high income countries (HICs) to the local specificities and realities of LMICs. This is a highly multidimensional problem starting from the limited support and integration of the rehabilitation services within the healthcare system, leading to suboptimal infrastructure and facilities and lack of integration within the cares (i.e., short hospitalization, lack of reimbursement of the rehabilitation sessions, lack of rehabilitation centers for outpatients rehabilitation). This problem is particularly relevant for patients suffering from neurological diseases, representing, however, a growing part of the patients and is responsible for the majority of the burden of the disability in LMICs (23). Here, specific neurorehabilitation services may be present in only two cities (Cotonou and Parakou).

Another limitation is the lack of information on EBP from health professionals themselves. Among the numerous barriers to EBP, the lack of specific academic training, which prevents rehabilitation specialists from comprehending the research language, is indeed one of the most significant obstacles in implementation of EBP in LMICs. Other factors currently limiting the knowledge translation include the lack of skills and the English language, motivation, and the ability to make decisions. Positive attitudes and motivation served as facilitators of transfers. Lack of time, lack of financial resources, limited access to scientific journals (despite program to support free access to publications), and applicability of research to rural settings were identified as organizational barriers (24).

## Call for action: The development of high-quality scientific evidence in rehabilitation in Benin

Different directions need to be taken simultaneously to drive sustainable changes.

Educational programs and dedicated workshops should be developed to inform the local healthcare professionals about the concept of level of evidence and clinical recommendations. The utilization of new affordable technologies (e.g., mHealth) may provide an alternative to the above-mentioned restrictions of rehabilitation or may supplement the present rehabilitation programs (13). mHealth has the potential to significantly benefit LMICs by overcoming a shortage of healthcare professionals, advancing rehabilitation, and reducing health inequalities through increased access to rehabilitation. Educational programs for rehabilitation professionals should be present in different regions of the country. In this context, the University of Parakou will launch a second educational program in physiotherapy (bachelor level) in Benin with the support of UHasselt. For neurorehabilitation particularly, a “summer school” has been established in Parakou, Benin, offering 12 courses and 2 weeks of workshop on mHealth and technology-supported solutions. In addition to this, a clinical internship of 3 months has also been initiated in collaboration with the Hasselt University, Belgium.

Despite these educational efforts, the lack of direct and easy translation of the validating studies performed in HIC may currently limit the applicability of EBP in LMICs. It is therefore necessary that evidence be developed locally by researchers in the country collaborating directly with researchers from HICs.

## Current collaborative activities

The Hasselt University and the University of Parakou signed a memorandum of understanding to strengthen the cooperation between the two institutions. The project is supported by both the rector and the vice rector of the University of Parakou for the cooperation between universities in Benin. Besides individual institutions, the collaboration is supported by the Ministry of Health and the Ministry of Education, thus showing the strong commitment of the country to further develop the quality of rehabilitation services. The bilateral collaboration also aims to expand the educational programs to include inland regions, such as central Benin in this sphere, too.

Based on collaborative research between UHasselt and UParakou in recent years, different axes of research and development have been identified to ease the implementation of the rehabilitation services in Benin. After mutual visits of (assistant) professors of both universities, 3 doctoral students were enrolled in a bilateral program including a 3-month research stay in Belgium yearly. The stay was focused to cover scientific training with the aim to perform data collection and interpretation in Benin. These activities could potentially lead to internationally respected results along with direct impact in the regional clinical and academic services as well.

In an attempt to further develop EBP in Benin, various research projects are currently underway. The first set of research are patient centric and focus on running qualitative studies to document the current (lack of) rehabilitation services that were attended during and after hospital discharge (14-day maximal inpatient stay). These studies were run in the subacute and chronic phases after an event of stroke. Patients' requirements and expectations about the rehabilitation were also evaluated, after which further studies need to investigate the level of acceptance of the different types of interventions (e.g., conventional rehabilitation sessions, group sessions, home exercises, and technological aids). As key players in the process, the healthcare professionals should be involved in this process, and therefore interviews, questionnaires, and focus groups also need to be performed to fully understand the needs of the clinicians. Currently, the modalities (i.e., types, volume, frequency, intensity, and supervision) and formats (individual or in group) of supervised exercise therapy as well as physical activity (PA) practice in community-dwelling chronic stroke survivors in Parakou are being explored. The patients' perceived barriers and facilitators to PA practice are also assessed. The content of physical therapy (PT) practice within inpatient rehabilitation facilities for stroke survivors in Benin, at the acute and sub-acute stages, is described using a standardized taxonomy (length of stay and total therapy time). These insights are believed to contribute to opening the “black-box” of stroke inpatient rehabilitation in Benin (25).

The initial results of these research lines will determine the extent to which interventions need to be adapted (e.g., infrastructure, equipment, professional education level, duration, intensity, patients' education, and cultural aspect) before being tested in the field (26). For example, the results of this first set of studies will be used to design interventions that are perfectly adapted to the situation of hospitalized and community-based subacute and chronic stroke survivors in Benin.

Other research focuses on the validation of the (modified) intervention. Pilot and feasibility studies are envisioned first, and later on validation is done in bigger studies (i.e., multicentric RCTs). At this moment, a feasibility and effectiveness study of an intensive rehabilitation exercise program at the acute stage of stroke is being prepared. The outcomes of this pilot study should guide the design of a randomized controlled trial comparing conventional care and an intensive exercise program at the acute phase of stroke on their clinical outcomes. Additionally, the feasibility and effects of a 10-week community-based PA program using the WalkWithMe app (mHealth) on walking performance in stroke survivors in Benin are also being investigated. WalkWithMe is a personalized mobile application that has been previously tested in Belgium in persons with multiple sclerosis to motivate patients to walk more during the rehabilitation process (27). In the future, a knowledge center for technology-supported rehabilitation is envisioned, including affordable technology and mHealth solutions that are not only applied in expertise centers but also allow independent use in community-located primary care centers, or even at home. In the future, Apps developed in UHasselt for neurorehabilitation, gait training, upper limb function, and cognitive-motor dual tasks will be transferred to Benin for cultural adaptation. New solutions for identified needs will be elaborated by local researchers from both the disciplines of human-machine interaction and rehabilitation (28).

It is strongly suggested that the discussion with the policymakers should start in parallel with the development of the scientific evidence to determine the best way to integrate this intervention in the care paths (and public health system). It is also significant to ensure the accessibility of these interventions to as many patients as possible, despite the limited budget. The consortium already had several meetings with the Beninese Ministry of Health to prepare this next step and to integrate technologies and mHealth solutions in the provision of care.

## Conclusion

Despite the level of evidence supporting various rehabilitation interventions still being sparse in HIC, developing strong local research capacity and knowledge in LMICs is the best solution to increase the availability of rehabilitation services and their implementation in the long term.

By developing local research and using culturally and locally adapted interventions, scientific evidence can be progressively built, thanks to the collaborations between the universities of Parakou and Hasselt. The ultimate result of this collaboration is expected to not only increase the availability of rehabilitation services, including technology-supported interventions, but also increase the quality of such services.

## Author contributions

BB wrote the first version of this paper. All authors contributed to the article and approved the submitted version.

## Conflict of interest

The authors declare that the research was conducted in the absence of any commercial or financial relationships that could be construed as a potential conflict of interest.

## Publisher's note

All claims expressed in this article are solely those of the authors and do not necessarily represent those of their affiliated organizations, or those of the publisher, the editors and the reviewers. Any product that may be evaluated in this article, or claim that may be made by its manufacturer, is not guaranteed or endorsed by the publisher.
